# Mini-extracorporeal Circulation and Off-pump Techniques Associated with Less Inflammatory Gene Expression as Compared to On-Pump in the 24-hour Postoperative Window Following Coronary Artery Bypass Grafting

**DOI:** 10.1186/1749-8090-10-S1-A101

**Published:** 2015-12-16

**Authors:** William T Brinkman, John J Squiers, Kyle R Covington, David A Wheeler, Mani Arsalan, Robert L Smith, Michael J Mack, J Michael DiMaio

**Affiliations:** 1 Department of Cardiac and Thoracic Surgery, The Heart Hospital Baylor Plano, Plano, TX, 75093, USA; 2 Department of Molecular and Human Genetics, Baylor College of Medicine, Houston, TX, 75030, USA

## Background/Introduction

Mini-extracorporeal circulation units (MECC) were developed to reduce inflammation (SIRS) associated with on-pump coronary bypass surgery (ONCAB) without the increased technical demands of off-pump surgery (OPCAB).

## Aims/Objectives

We sought to compare the inflammatory response induced by all three techniques (OPCAB, MECC, ONCAB) using gene expression analysis techniques.

## Method

Patients (n = 102) undergoing isolated coronary bypass grafting were prospectively enrolled and divided into cohorts of OPCAB (n = 34), MECC (n = 34), and ONCAB (n = 34). Serial blood samples were collected at multiple time points (preoperatively, at skin closure, and postoperatively at 24 and 48 hours). Total RNA was isolated from all samples, and gene expression analyses were performed with the Illumina HumanHT12 v4 microarray. Individual samples were randomly allocated into training (n = 149) and test (n = 141) sets to validate results.

## Results

Randomization between the training and test set showed no significant differences in clinical parameters between the two sets. Supervised gene expression analyses indicated that inflammatory pathways were significantly increased in all treatments, and these pathways were increased in ONCAB vs MECC or OPCAB, with no difference between MECC and OPCAB, at 24 hours postoperatively by multiple testing approaches. By 48 hours postoperatively, differences in inflammatory gene expression were no longer detectable.

## Discussion/Conclusion

In the acute 24-hour period following surgery, inflammatory expression does appear to be less in MECC compared to ONCAB and no different in MECC compared to OPCAB. However, given that this difference resolved quickly, the clinical significance of this result is unclear. Our analysis will guide further investigation into the inflammatory response induced by OPCAB, MECC, and ONCAB techniques.

